# The correlation between atmospheric visibility and influenza in Wuxi city, China

**DOI:** 10.1097/MD.0000000000021469

**Published:** 2020-08-07

**Authors:** Juan Liu, Enpin Chen, Qi Zhang, Ping Shi, Yumeng Gao, Yujun Chen, Wendong Liu, Yiran Qin, Yuan Shen, Chao Shi

**Affiliations:** a Wuxi Center for Disease Control and Prevention, Wuxi; b Jiangsu Center for Disease Control and Prevention, Nanjing, Jiangsu, China; c Westminster College, Salt Lake City, UT.

**Keywords:** atmospheric visibility, distributed lag non-linear model, influenza, influenza like illness

## Abstract

Influenza is an acute respiratory infectious disease that poses a threat to public health. We assessed the association between atmospheric visibility and influenza and influenza-like illness (ILI) in Wuxi city, China.

Daily meteorological data, ILI activity, and influenza virus infection rates were collected between 31 December 2012 and 31 December 2017. A distributed lag non-linear model (DLNM) was used to analyze the exposure-lag-response of ILI and influenza activity and daily average visibility.

A total of 12,800 cases were detected; 1046 cases (8.17%) were of Flu-A and 527 (4.12%) were of Flu-B infection. Our analysis suggested a non-linear relationship between atmospheric visibility and influenza: U-shaped for ILI, and L-shaped for Flu-A and Flu-B. Comparing low visibility (2.5 km) to ILI cases, the risk appeared between day 1 and day 2. For Flu-A, the risk appeared between days 5 and 9, whereas for Flu-B, the risk effect was much stronger and had a longer reaction delay, staying above zero until day 9. The protective effects of high visibility (14 km) on ILI and Flu-B occurred the same day or one day later. However, we found no association between high visibility and Flu-A.

In conclusion, our study contributes novel evidence for the effects of atmospheric visibility on influenza. These findings are important for the development of influenza surveillance and early warning systems in Wuxi city.

## 1 Introduction

Influenza is an acute respiratory infectious disease, which poses a great threat to public health, resulting in substantial morbidity and mortality each year.^[1]^ It is a contagious disease primarily caused by the influenza viruses. The influenza A (Flu-A) and B (Flu-B) viruses are the primary causes of this acute viral respiratory disease. Influenza like illness (ILI) is clinically characterized by a common group of symptoms that may be caused by the influenza virus or other pathogens, such as the respiratory syncytial virus and parainfluenza virus.^[2]^ In south China, the activity of Flu-A viruses usually peaks twice; once in summer (July-September) and once in winter (January–March). Flu-B is predominant during winter (December–April).^[3–5]^ The seasonality of influenza may depend on a variety of factors, including seasonal host health, socioeconomic level, subtype of influenza virus, and environmental factors.^[6]^

Atmospheric visibility is a proven indicator of ambient air quality.^[7]^ Moreover, visibility provides a useful proxy for the assessment of environmental health risks from ambient air pollutants, and is useful for the assessment of the impact of air pollution on public health.^[8]^ Loss of visibility is easily measured and arises from a loss of contrast between an object and the background, and the attenuation of the light signal from an object due to the scattering and absorption of light by fine particulates (e.g., PM2.5 and PM10) and gaseous pollutants.^[9,10]^ Loss of visibility is regarded as a primary indicator of ambient air quality in urban areas.^[11]^ Air pollution has been well documented as a major public health issue worldwide, and a growing body of epidemiological and clinical evidence has shown that pollutants increase the risks of numerous diseases,^[12–15]^ including the incidence of air pollution-related diseases, such as stroke, ischemic heart disease, and respiratory infections.^[16]^ Several reports have indicated that indoor air pollution is associated with acute lower respiratory tract infections.^[17–19]^ Moreover, numerous studies have confirmed that exposure to air pollutants is closely associated with the localized transmission of influenza.^[20]^

Until now, research has focused on the impacts of climate change on influenza,^[21–23]^ but rarely has the relationship, especially the exposure-lag-response, between the activity of various types of influenza viruses and air pollution (as measured by atmospheric visibility) been studied. To redress this deficiency, we attempted to use distributed lag non-linear models (DLNM) along with disease surveillance and laboratory data for Wuxi city to elucidate the influence of atmospheric visibility on ILI and on different subtypes of the influenza virus.

## 2 Methods

### 2.1 Population and study area

Wuxi is a modern city with an area of 4627 square kilometers and a population of 4.93 million as of 2017 (Wuxi municipal bureau of statistics). The city lies in the southeast of Jiangsu province which has a sub-tropical maritime climate with clear-cut seasonal changes. It has high temperatures and high relative humidity in summer and cold temperatures with little precipitation in winter.

### 2.2 Data collected

Influenza-like Illness (ILI) is defined as the existence of a fever (≥38°C) and cough and/or sore throat in the absence of a known cause other than influenza. In our study, ILI (%) refers to the percentage of all outpatient visits for ILIs, as automatically identified by the hospital information systems (HIS) of 4 sentinel hospitals (two municipal hospitals and two primary hospitals). Data on at least 40 cases of ILIs were collected weekly from the 4 hospitals. This included patients who had not received antiviral treatment and for whom onset of symptoms had occurred within the last three days. The specimens were analyzed in laboratories for influenza viruses and subtyped using the real-time fluorescent quantitative polymerase chain reaction assay.

We collected the meteorological data from 31 December 2012 to 31 December 2017. This data included visibility (km), average relative humidity (%), and rainfall, and was obtained from Wuxi municipal meteorological service center. The number of ILI cases and other ILI data were then standardized as ILI per 10,000 outpatient visits. Influenza activity was calculated by multiplying the weekly positive rates of influenza virus A or B by the daily number of ILI cases. The study was passed by Ethics Committee of Wuxi Center for Disease Control and Prevention.

### 2.3 Statistical analysis

The relationship between exposure to meteorological factors or air pollution and the activity of influenza virus in the population is nonlinear and the association always lasts well beyond the exposure period.^[24,25]^ Gasparrini et al established a statistical framework called distributed lag non-linear model (DLNM). DLNMs are based on the cross-basis function, and model the nonlinear exposure-response and the lag structure of a relationship simultaneously.^[26,27]^ In our research, a DLNM was used to explore the potential exposure-lag-response association between daily average visibility, ILIs, and positive influenza virus samples. To achieve our study purpose, a time series model assuming Quasi-Poisson distribution was fitted. A cross-basis matrix of visibility (cb[Vmean]) was included to explore the cumulative and delayed effects of the daily average visibility. In order to adjust for the long-term trend, a smooth function of time (Timet) was taken into account in the model. Daily average relative humidity (Rhmean) and daily average rainfall were weather-related confounders.^[28,29]^ Day of the week (DOWt) was also incorporated into the final model to control for the effect of potential confounding factors. The statistical model used in our study was defined as follows: 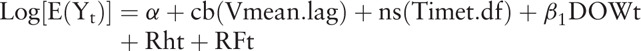


The cross-basis matrix of visibility was built by using a nature cubic spline with 3 df for the space of visibility and 3 df for the log scale of lag spaces. The days of lag structure in the model were determined by the incubation period of influenza; we chose a lag of 10 days in our final models.^[23]^ We analyzed the data using R, and the “dlnm” package in R was used to conduct the statistical analysis of exposure-lag-response effects.

## 3 Results

### 3.1 General characteristics

**Figure 1:**
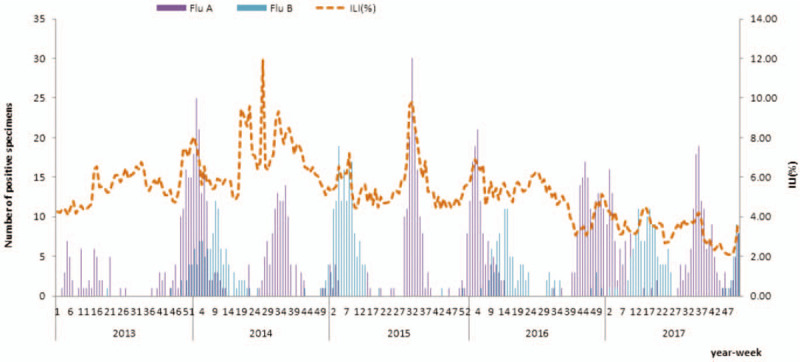
ILI and the number of specimens positive for influenza by subtype in Wuxi city.

During the period from 31 December 2012 to 31 December 2017, a total of 12,800 specimens were analyzed in the laboratory; 1573 (12.28%) were positive for the influenza virus, of which 1,046 (8.17%) were typed as Flu-A and 527 (4.12%) as Flu-B. ILIs (%) and Flu-A peaked twice each influenza year, with a first wave occurring in the summer and another one in the winter. Flu-B cases occurred frequently in winter or early spring (Fig. 1). The variables visibility, relative humidity, rainfall, ILIs(‱(One ten thousandth)), Flu-A, and Flu-B are presented on the scale of every 10,000 outpatients (Table 1).

**Table 1:**
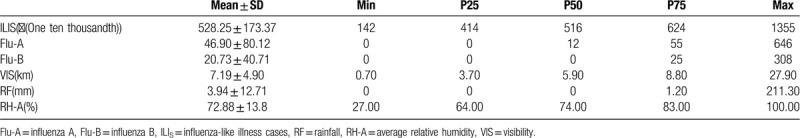
The characteristics of visibility, ILI_S_ and confirmed influenza cases every 10,000 outpatient visits during 2013 to 2017 in Wuxi city.

### 3.2 Risk response to visibility

**Figure 2:**
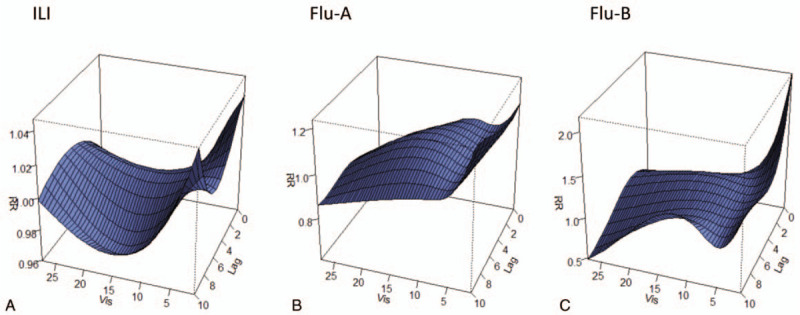
Exposure-lag-response surface for ILI, Flu-A, and Flu-B in Wuxi city.

**Figure 3:**
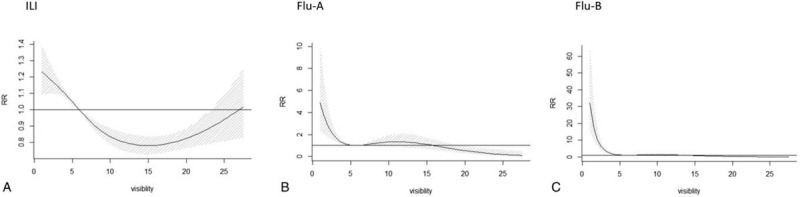
Cumulative association and visibility distribution of ILI, Flu-A, and Flu-B in Wuxi city (5.9 km as reference).

The overall cumulative association analysis suggested a non-linear relationship between atmospheric visibility and influenza. The relationship was U-shaped for ILI, and L-shaped for Flu-A and Flu-B (Figs. 2 and 3). Using 5.9 km (P_50_) as a reference, we found that the visibility of <5.9 km increased the risk of influenza, and this risk decreased as the value of visibility increased, with a time delay. The risk of low visibility was stronger for Flu-B when compared with ILI and Flu-A.

**Figure 4:**
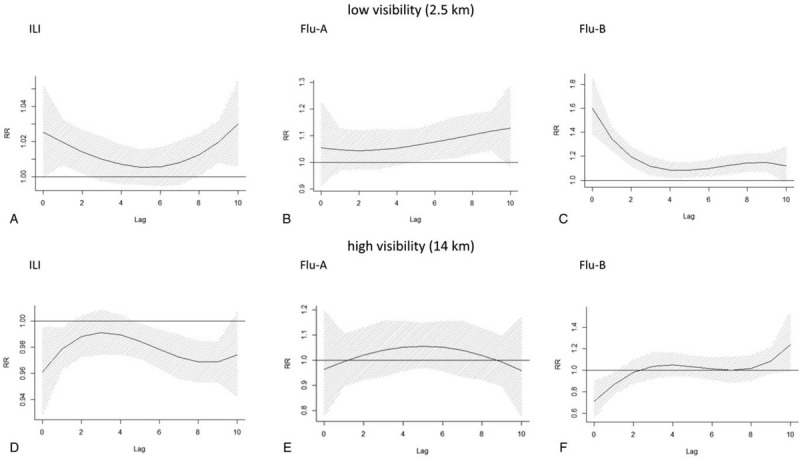
Lag-response association at low/high visibility of ILI, Flu-A, and Flu-B in Wuxi city.

Based on the results above, we then analyzed the delay in the association between low/high visibility and influenza related outcomes (Fig. 4). The low value for visibility was selected using the 10th percentile for visibility (2.5 km). Low visibility was associated with an increased number of ILI cases (lag_1_: RR = 1.019, 95% *CI* = 1.007–1.032) with a one-day delay (lag_2_: RR = 1.014, 95% *CI* = 1.002–1.026) (Fig. 4A). For Flu-A, the risk effects of low visibility appeared at day 5, with a delay till day nine (lag_5_: RR = 1.063, 95% *CI* = 1.005–1.125; lag_9_: RR = 1.117, 95% *CI* = 1.045–1.193) (Fig. 4B). The effect of low visibility for Flu-B risk was much stronger and had longer reaction delay, staying above zero until day 9 ((lag_0_: RR = 1.600, 95% *CI* = 1.383–1.852; lag_9_: RR = 1.146, 95% *CI* = 1.074–1.223) compared with ILI and Flu-A (Fig. 4C).

The high value for visibility was selected based on the 90th percentile for visibility (14 km). For ILI and Flu-B, high visibility conferred a protective effect the same day and 1 day later (lag_0_: RR (ILI) = 0.961, 95% *CI* = 0.928–0.995; RR (Flu-B) = 0.711, 95% *CI* = 0.561–0.901; lag_1_: RR (ILI) = 0.979, 95% *CI* = 0.963–0.995; RR (Flu-B) = 0.871, 95% *CI* = 0.775–0.978) (Fig. 4D and E). However, we found no association between high visibility and Flu-A (Fig. 4F).

## 4 Discussion

In this study, we aimed to explore the potential linkage between atmospheric visibility and human influenza incidence. To our knowledge, this is the first study to analyze the relationship between ILI, the subtypes of influenza virus, and atmospheric visibility in China. We found that low visibility (2.5 km) was associated with ILI and influenza A/B incidence. Our study suggests that exposure to low atmospheric visibility was a contributing factor to the risk of influenza infection. The possible mechanisms for this association between low visibility and influenza are complex. It is likely that low visibility may cause host defense disorders, including both innate and cell-mediated immune responses against bacterial and viral infections.^[30,31]^ On days of low visibility, fine particles have the ability to transmit influenza viruses, increasing the chance of transmission of the virus. Fine particles with viruses attached can also be inhaled resulting in the direct delivery of the viral agents to the respiratory epithelial cells.^[32,33]^ Various experimental studies have suggested that the deposition of particles on the epithelial cells that line the airways activates inflammatory signaling cascades.^[34,35]^ Other studies have reported that exposure to PM_2.5_ is associated with dysfunction of the pulmonary tracheal cilia and decreased activity of alveolar macrophages, which in turn may enhance an individual's susceptibility to viral agents.^[36,37]^

We also found that the effect of low visibility on ILI was subject to a delay of 1 to 2 days. Low visibility was associated with an increase in the daily number of Flu-A cases at days 5 to 9. The risk association between low visibility and Flu-B was much stronger and had a longer reaction delay, staying above zero until day 9. This serves as a reminder that the effect of low visibility on ILI tends to be acute. In contrast, the effect of low visibility on Flu-A tended to be slow in taking effect and the effect on Flu-B tended to be acute, with a longer delay. Several previous studies investigating the lag effect of air pollutants on respiratory diseases showed similar results. In a study of the association between air pollution and the daily number of consultations due to upper respiratory tract infections in a general outpatient clinic in Hong Kong, the lag times ranged from 0 to 3 days.^[38]^ Chen et al also found that increased ambient PM_2.5_ concentrations are associated with incident cases of influenza at a lag time of 2 to 3 days.^[20]^ In a time-series study in Brisbane, Australia, associations between exposure to air pollution and the incidence of pediatric influenza were reported to occur at a longer lag time of 10 days.^[39]^ The differences in these reported results may also be attributed to the difference between influenza and ILI. The results showed that high visibility (14 km) was protective against ILI and Flu-B infection, with the protective effect occurring the same day and 1 day later. The lag time in the association between atmospheric visibility and influenza activity was also identified, which can be very helpful for better understanding the mechanisms underlying this association and for the control and prevention of influenza. This information could potentially be used as part of the surveillance system for ILI and various influenza subtypes, for an integrated strategy for infectious disease control and prevention.

A reduction in daily visibility is a widespread and increasing problem, which is associated with a deterioration in air quality. In the absence of air pollutant data, the use of visibility measures can be deployed quickly by environmental health authorities for estimating the health effects of air pollution, including the shape of exposure–response curves for influenza. This locally generated evidence can provide important support for public health through air quality improvement policies, particularly because of its potential to facilitate the protection of the health of susceptible subgroups in the population, such as children and elderly people.^[40]^

There are some limitations to our study. First, we did not have information on patient characteristics, such as age and sex, which tend to be related to influenza incidence. Second, some individuals with influenza or ILI may not have sought medical attention, and therefore, could not be considered in our surveillance data. Adults and older people usually prefer self-medication, which might have led to selection bias in our study. Third, the prevalence of influenza can also be related to viral activity, the immunity status of the population, and social factors. However, in this study, we have focused solely on the relationship between atmospheric visibility and influenza activity, and thus, the other factors need further analysis in future studies.

In conclusion, the present study contributes novel evidence regarding the effects of atmospheric visibility on influenza activity in Wuxi city. These findings may be useful and important for the development of influenza surveillance and early warning systems. More extensive studies are needed in order to establish the relationships between fine particles and influenza activity in regions of China with varying degrees of pollution. Further laboratory studies are also needed in order to understand the plausible mechanisms underlying this association.

## Acknowledgments

The authors are thankful to the support from Wuxi People's Hospital, Wuxi Children's Hospital, Jiangyin People's Hospital and Jiangyin Shanguan Hospital.

## Author contributions

**Conceptualization:** Juan Liu, Enpin Chen.

**Data curation:** Juan Liu, Qi Zhang.

**Investigation:** Ping Shi, Yumeng Gao, Yujun Chen, Yiran Qin.

**Methodology:** Juan Liu, Qi Zhang, Wendong Liu.

**Project administration:** Yuan Shen, Chao Shi.

**Writing – original draft:** Juan Liu.

**Writing – review & editing:** Juan Liu, Yuan Shen, Chao Shi.

All authors approved the final version of the paper.
